# Wellbeing in Winter: Testing the Noticing Nature Intervention During Winter Months

**DOI:** 10.3389/fpsyg.2022.840273

**Published:** 2022-04-25

**Authors:** Holli-Anne Passmore, Alissa Yargeau, Joslin Blench

**Affiliations:** Department of Psychology, Concordia University of Edmonton, Edmonton, AB, Canada

**Keywords:** wellbeing, Noticing Nature Intervention, winter, hope, connectedness

## Abstract

The main objective of this 2-week RCT study was to test the efficacy of the previously developed Noticing Nature Intervention (NNI) to boost wellbeing during winter months. The NNI consists of noticing the everyday nature encountered in one’s daily routine and making note of what emotions are evoked. Community adults (*N* = 65) were randomly assigned to engage in the NNI or were assigned to one of two control conditions. Paired *t*-tests revealed significant increases pre- to post-intervention in the NNI group for positive affect (*d* = 0.43), elevation (*d* = 0.59), nature connectedness (*d* = 0.46), and hope agency (*d* = 0.64), and a marginally significant increase in transcendent connectedness (*d* = 0.41). No significant pre-post difference emerged for any aspect of wellbeing in the control conditions. Analysis of qualitative findings revealed that negative emotion themes were 2.13 times more likely to be associated with built photos than with nature photos. Feelings of peace, awe, happiness, humbleness, and hope were more likely to be associated with nature photos, while feelings of annoyance, loneliness, curiosity, uncertainty, anger, yearning, and comfortableness were more likely to be associated with built photos. Overall, results indicated that engaging in the NNI can provide a wellbeing boost, even in the cold of winter. This study is the first (to our knowledge) to test any nature-based wellbeing intervention during colder, winter months, and to directly assess the impact of a nature-based wellbeing intervention on levels of hope.

## Introduction

“*It was a beautiful, blue and frozen morning…and Saturday, too! The sun was bright and the old snow sparkled. The day was young and full of opportunities!*” (Participant jmhd9423)

Within a positive psychology framework, wellbeing is construed as consisting of aspects of hedonia, such as positive emotions, satisfaction with life, and happiness (Keyes, 2002; Diener, 2009), and eudaimonia, including meaning in life and self-transcendence (see Huta and Waterman, 2014). Evidence is emerging that engaging with nature is an important pathway to boosting both these facets of wellbeing. For example, findings from large population-based studies have consistently demonstrated that people experience greater wellbeing when in more natural, compared to more built, environments (e.g., MacKerron and Mourato, 2013; White et al., 2013). Literature reviews of the benefits of connecting with nature (Capaldi et al., 2015), in addition to systematic reviews of exposure to nature (Tillmann et al., 2018) and nature-based interventions (Coventry et al., 2021), all demonstrate that connecting with nature boosts wellbeing. Systematic reviews and meta-analyses of randomized controlled trials (RCT) of brief nature-interventions also evidence the important role that engaging with nature has on mental wellbeing (McMahan and Estes, 2015; Hunt et al., 2021). Indeed, engaging with nature has been posited to be a basic psychological need (Searles, 1960; Baxter and Pelletier, 2019; Hurley and Walker, 2019).

Despite this evidence, research validating nature-based wellbeing interventions is somewhat sparse. Most RCTs in this area involve brief, one-time sessions of exposure to or engagement with nature wherein wellbeing is measured immediately after the engagement. Some exceptions, though, do exist. Two such exceptions are studies which involved participants noting three good things in nature on a daily basis for 1 week (McEwan et al., 2019; Pocock et al., 2021, under review). In both of these studies, participants randomly assigned to the Three Good Things in Nature condition reported higher levels of wellbeing. Another two exceptions are studies by Passmore and colleagues testing the Noticing Nature Intervention (Passmore and Holder, 2017; Passmore et al., 2022). The Noticing Nature Intervention consists of being mindful, on a daily basis for 2 weeks, of the emotions that are evoked by the everyday nature one encounters in their everyday routine. Both of Passmore and colleagues’ experimental studies involved random assignment to either the Noticing Nature Intervention or a control condition. In both studies, at the end of 2 weeks, wellbeing was significantly higher in the Noticing Nature Intervention condition than in the control conditions (*d*s from 0.49 to 0.69). While such research is slowly becoming more common, gaps still exist in the extant literature.

### Research Gaps

Nature-based interventions have thus far been primarily conducted in spring, summer, or early fall months when weather tends to be mild or pleasant, and vegetation growth is colorful and green. To our knowledge, no experimental, RCT nature-based wellbeing intervention studies—of either short or longer duration—have been conducted in colder winter months.

There is also a relative dearth of research examining engaging with nature as a pathway to hope. Empirical findings do, though, suggest that people find hope in nature (Passmore and Howell, 2016). For example, in a qualitative study involving discussions and autobiographical writing exploring sources of hope, Hicks (1998) reported that the natural world emerged first in a collective list of 10 sources of hope. Berger and McLeod (2006) identified increased hope as an outcome associated with counselling approaches in which clients’ connectedness to nature is explored; Turner and Cox (2004) found that when participants were asked to take photos of what inspired hope in them, photos of nature were common. Lastly, in Passmore and colleagues’ experimental RCT studies (Passmore and Holder, 2017; Passmore et al., 2022), a theme of hope emerged from the qualitative writings of participants who had been randomly assigned to engage in the Noticing Nature Intervention. However, despite this empirical evidence, no experimental studies have specifically assessed the impact of engaging with nature on hope.

### The Current Study

Given these research gaps, the primary objective of the current study was to test the efficacy of the Noticing Nature Intervention (Passmore and Holder, 2017) to enhance wellbeing during winter months. A secondary objective was to assess boosts in hope, as a specific aspect of wellbeing, as a direct result of engaging in the Noticing Nature Intervention. Our last objective was to test the generalizability of Passmore and colleagues’ previous findings (Passmore and Holder, 2017; Passmore et al., 2022) regarding the wellbeing benefits of engaging in the Noticing Nature Intervention beyond an undergraduate population sample.

## Materials and Methods

### Recruitment and Participants

Participant recruitment and participation occurred from January to mid-March in 2021. Two recruitment methods were used: hand-delivered flyers to residences in Edmonton, Alberta, Canada, and word-of-mouth *via* social media contacts. The recruitment poster noted that participants were wanted who were “interested in participating in research examining the relationship between taking digital photos and emotional experiences in winter.” While the ad noted that the researchers were interested in emotional reactions to everyday surroundings, no indication of the type of those surroundings was given. That is, no mention of nature was made. This was done to avoid initial self-selection bias of participants who had greater affiliation with nature. A total of 91 participants were recruited, 65 of whom completed all parts of the study. Of these 65, 43 were from Edmonton, and the remaining 21 were from other cities in Canada or the northern USA. The mean age of participants was 46.75 years (SD = 13.60, range: 21–75, Median = 47.00); 51 participants were female, 14 were male. Participants’ occupations varied and included teacher/university professor, business administration or management, fine arts performer, mental health professional, university student, retired, and unemployed (see Table 1 for complete details). Mean age was similar across all conditions, as was ratio of female to male participants, and the general spread of occupations.

**Table 1:** Participant occupations.

Occupation	Count
Educator (Teacher or Professor)	11
Retired	11
Business: Manager	5
University Student	5
Unemployed	5
Business: Administration	4
Fine Arts: Artist, Musician, Theatre	4
Trades	3
Psychologist	2
Social Work	2
Banker	1
Clergy	1
Ecologist	1
Editor	1
Information Technology	1
Marketing	1
Engineer	1
Nurse	1
Physician	1
Policy Advisor	1
Scientist	1
Not reported	2

### Measures

#### Hedonic Well-Being

##### Positive and Negative Affect

The 20-item Positive and Negative Affect Scale (PANAS; Watson et al., 1988) includes 10 words pertaining to positive emotions (e.g., strong, enthusiastic) and 10 words pertaining to negative emotion (e.g., irritable, guilty). Respondents rate each item on a 5-point scale from 1 (*very slightly or not at all*) to 5 (*extremely*) to indicate the extent to which they experienced each of the listed emotions over the past 2 weeks. Positive affect (*α* = 0.92) and negative affect (*α* = 0.89) were calculated.

##### Satisfaction With Life

The Satisfaction with Life Scale (SWLS; Diener et al., 1985) is a five-item scale that measures life satisfaction. Participants rate items (e.g., the conditions of my life are excellent) on a 7-point scale ranging from 1 (*strongly disagree*) to 7 (*strongly agree*). Cronbach’s α was 0.89.

#### Eudaimonic Well-Being

##### Meaning in Life

Sense of meaning was assessed with Huta and Ryan’s (2010) Sense of Meaning Scale (SMS). This scale’s 12 items are either words or phrases that pertain to elements of meaning and purpose in life (e.g., meaningful, full of significance). Using a 7-point Likert scale with endpoints of 1 (*not at all*) to 7 (*extremely*), respondent rate the degree to which each item describes how they typically felt about their activities and experiences over the past 2 weeks. Huta and Ryan’s principal component analyses showed that a sense of meaning was a distinct aspect of well-being. Cronbach’s *α* in the current study was 0.96.

##### Transcendent Connectedness

Six items were selected from the Metapersonal Self (MPS) Scale (DeCicco and Stroink, 2007) to assess the extent to which an individual feels connected to wider aspects of humankind and life in general. Items (e.g., “My sense of identity is based on something that unites me with all other people” and “I see myself as being extended into everything else”) are rated on a 7-point scale with end points of *1 = Strongly Disagree* and *7 = Strongly Agree*. Cronbach’s *α* was 0.91.

##### Elevation

Huta and Ryan’s (2010) Elevating Experiences Scale (EES) is a 13-item scale which assesses a variety of emotions (e.g., inspired, elevated, deeply appreciative, profoundly touched, emotionally moved). Items are rated using a 7-point scale with endpoints 1 (*not at all*) and 7 (*extremely*), according to the degree to which each item describes how the respondent typically feels. Cronbach’s *α* was 0.97.

#### Hope

We used two measures of hope. The Herth Hope Index (HHI: Herth, 1992) was developed to capture the multidimensionality of hope within a shorter-form assessment tool. The 12 items of this scale (e.g., “I believe that each day has potential”) are rated on a scale from 1 (*strongly disagree*) to 4 (*strongly agree*). Cronbach’s *α* was 0.90. The Adult Hope Scale (AHS; Snyder, 2002) was developed within the context of Snyder’s Hope Theory, which defines hope as a capability to derive pathways to desired goals (i.e., hope pathways), and motivation and capability to use those pathways (i.e., hope agency). Two subscales are composed of four pathway items (*α* = 0.97; e.g., “I can think of many ways to get out of a jam”) and four agency items (*α* = 0.93; e.g. “I energetically pursue my goals”) which are rated using an 8-point scale ranging from 1 (*definitely false*) to 8 (*definitely true*).

#### Nature Connectedness

Two measures were used to assess nature connectedness. The single-item Inclusion of Nature in Self Scale (INS; Schultz, 2001) is rated by choosing one of seven diagrams depicting increasing degrees of overlap between a circle labeled “Self” and one labeled “Nature.” Mayer and Frantz’s (2004) Connectedness to Nature Scale (CNS) is composed of 14 items which assess a sense of oneness with the natural world (e.g., “I often feel a sense of oneness with the natural world around me”). Items are rated on a 5-point scale with endpoints 1 (*strongly disagree*) and 5 (*strongly agree*). Cronbach’s *α* was 0.87.

#### Time in Nature

At the end of the study, participants were asked to estimate how much time they had spent in nature per day, on average, over the course of the previous 2 weeks.

### Procedure

Participants went to the study website where they watched a brief 5-min video in which the main author provided an overview of the study. Those interested in continuing proceeded to the next page where they were presented with the consent form and asked to provide their email address. An email was sent to each participant with a link to Part 1 of the study, along with a randomly-generated participant ID code so that responses were not linked to email addresses to preserve anonymity. In Part 1, participants completed measures of well-being, hope, and nature connectedness, and demographic questions regarding age, gender, and occupation.

Following the procedure and instructions of the first study testing the Noticing Nature Intervention (Passmore and Holder, 2017), participants were then randomly assigned to one of three conditions—Nature, Built, or Delay (a business-as-usual control condition)—which constituted Part 2 of the study. Those assigned to the Nature condition (*n* = 24) were instructed to notice and be mindful, for the next 2 weeks, of how the natural elements and objects they encountered in their daily routines made them feel. (In essence, participants were asked to engage in the Noticing Nature Intervention.) As in the 2017 study, participants were also asked to take, and upload, digital photos of the nature scenes/objects that evoked emotions in them along with a brief description of the emotions that were evoked. A minimum of 10 photos spaced over the course of the 2 weeks was requested. It was stressed that the researchers were not concerned with the photos *per se* (e.g., quality, creativity), but rather with the participants’ emotional experience and reaction to how the objects/scenes made them feel.

Those assigned to the Built condition (*n* = 19) were provided the same instructions, except that they were to pay attention to how the human-built objects and scenes they encountered made them feel. Accordingly, these participants were asked to take/upload photos of human-built objects and scenes along with a brief description of the emotions evoked. During the 2-week intervention, participants in the Nature and Built conditions received reminder emails about the study activity (as per their random assignment) every day, along with a link to upload their photos and descriptions of emotions evoked.

As per Passmore and Holder’s (2017) study, a business-as-usual control condition (*n* = 22) was also utilized wherein participants were instructed to continue with their regular routine for the next 2 weeks, at the end of which they would be provided with instructions for the “emotional photography” portion of the study. In actuality, these participants merely completed the post-questionnaires in 2 weeks’ time and were then debriefed.

In 2 weeks’ time, all participants, regardless of condition, received an email with a link to the study’s website which they were asked to log-in to within 48 h to complete Part 3 of the study. Part 3 consisted of completing the same measures of well-being, hope, and nature connectedness which they had completed 2 weeks prior. All participants were debriefed, thanked, and given the opportunity to download the instructions for the Noticing Nature Intervention. All participants were given the opportunity to withdraw their responses (even though responses were anonymous). They were then directed to a separate website where they entered their email address for a chance to enter a draw for one of fifty $50 Amazon e-vouchers.

## Quantitative Results

Given the relatively small number of participants we were able to recruit in each condition for this study, we did not have sufficient power to conduct ANCOVAs or between-group *t*-tests to meaningfully examine between-group differences.^1^ Rather, we conducted within-group paired *t*-tests to examine if significant changes occurred within each group on levels of wellbeing, hope, and nature connectedness. Although still under-powered, our sample size was less-so for this type of analysis; nonetheless, these findings should be viewed with some caution and as preliminary. Significant differences from pre- to post-intervention in wellbeing, hope, and nature connectedness emerged in the Nature group. Participants in the Nature condition reported significantly higher levels of positive affect (*d* = 0.47), satisfaction with life (*d* = 0.43), elevation (*d* = 0.59), hope agency (*d* = 0.64), and nature connectedness (on the INS measure: *d* = 0.46), and marginally significantly higher transcendent connectedness (*d* = 0.41). Negative affect, a sense of meaning, and hope pathways did not differ significantly from pre- to post-intervention. In the Built and Delay conditions, no significant differences emerged for any measure of wellbeing, hope, or nature connectedness from pre- to post-intervention (*p*s > 0.134). See Tables 2 and 3 for full statistics.

**Table 2:** Descriptive statistics.

DV*n* (each condition)	Delay		Human-Built		Nature	
	*M* (SD)		*M* (SD)		*M* (SD)	
Positive affect	post:	35.25 (5.92)	post:	33.00 (7.33)	post:	34.39 (7.79)
*n* = 20, 19, 23	pre:	34.30 (5.85)	pre:	30.79 (6.92)	pre:	31.48 (8.88)
Negative affect	post:	17.23 (6.22)	post:	18.16 (6.18)	post:	19.67 (7.90)
*n* = 22, 19, 24	pre:	17.96 (5.80)	pre:	20.53 (6.60)	pre:	20.79 (8.36)
Satisfaction with life	post:	26.59 (5.46)	post:	23.63 (6.86)	post:	25.92 (6.91)
*n* = 22, 19, 24	pre:	27.05 (4.94)	pre:	22.16 (7.15)	pre:	24.71 (6.91)
Meaning	post:	57.25 (14.41)	post:	52.63 (15.12)	post:	53.26 (15.73)
*n* = 21, 16, 23	pre:	55.81 (14.69)	pre:	50.31 (14.70)	pre:	48.96 (17.51)
Transc. Co.	post:	31.36 (6.88)	post:	31.63 (5.90)	post:	31.08 (8.16)
*n* = 22, 19, 24	pre:	30.64 (7.93)	pre:	29.68 (6.77)	pre:	28.83 (7.96)
Elevation	post:	57.09 (18.90)	post:	58.61 (14.21)	post:	62.29 (20.66)
*n* = 21, 18, 24	pre:	55.52 (17.69)	pre:	54.22 (16.02)	pre:	50.04 (21.58)
Hope (HHI)	post:	39.86 (5.34)	post:	37.95 (5.46)	post:	38.21 (5.79)
*n* = 21, 19, 24	pre:	39.38 (5.69)	pre:	37.58 (5.65)	pre:	37.29 (7.09)
Hope-Path. (AHS)	post:	25.41 (4.80)	post:	25.11 (4.00)	post:	24.71 (5.65)
*n* = 22, 19, 24	pre:	25.18 (5.63)	pre:	24.68 (4.02)	pre:	23.88 (6.33)
Hope-Agency (AHS)	post:	26.67 (5.07)	post:	24.05 (4.43)	post:	25.46 (5.50)
*n* = 21, 19, 24	pre:	25.67 (5.46)	pre:	23.74 (4.86)	pre:	23.58 (6.66)
Nat. Con. (INS)	post:	4.05 (1.28)	post:	5.26 (1.45)	post:	4.33 (1.66)
*n* = 21, 19, 24	pre:	3.86 (1.35)	pre:	5.16 (1.46)	pre:	3.96 (1.68)
Nat. Con. (CNS)	post:	71.00 (13.57)	post:	77.42 (12.53)	post:	74.41 (14.32)
*n* = 22, 19, 22	pre:	72.68 (11.48)	pre:	75.53 (13.57)	pre:	73.05 (12.80)

Transc. Co., Transcendent Connectedness; HHI, Herth Hope Index; Hope-Path., Hope Pathways; AHS, Adult Hope Scale; Nat. Con., Nature Connectedness; INS, Inclusion of Nature in Self; CNS, Connectedness to Nature Scale.

**Table 3:** Paired *t*-tests.

	Delay	Human-Built	Nature
Positive affect	*t*(19) = 1.04, *p* = 0.310 *d* = 0.23 [−0.21, 0.68]	*t*(18) = 1.51, *p* = 0.148 *d* = 0.35 [−0.12, 0.81]	*****t***(22) = 2.26, ***p*** = 0.034** *****d*** = 0.47 [0.04, 0.90]**
Negative affect	*t*(21) = −0.66, *p* = 0.520 *d* = −0.14 [−0.56, 0.28]	*t*(18) = −1.49, *p* = 0.153 *d* = −0.34 [−0.80, 0.13]	*t*(23) = −0.96, *p* = 0.347 *d* = −0.20 [−0.60, 0.21]
Satisfaction with life	*t*(21) = −0.58, *p* = 0.571 *d* = −0.12 [−0.54, 0.30]	*t*(18) = 1.24, *p* = 0.230 *d* = 0.29 [−0.18, 0.44]	*****t***(23) = 2.13, ***p*** = 0.044** *****d*** = 0.43 [0.01, 0.85]**
Meaning	*t*(20) = 0.79, *p* = 0.441 *d* = 0.17 [−0.26, 0.60]	*t*(15) = 0.66, *p* = 0.521 *d* = 0.16 [−0.33, 0.66]	*t*(22) = 1.26, *p* = 0.221 *d* = 0.26 [−0.16, 0.68]
Transcendent. connectedness	*t*(21) = 0.91, *p* = 0.373 *d* = 0.19 [−0.23, 0.61]	*t*(18) = 1.91, *p* = 0.073 *d* = 0.44 [−0.04, 0.90]	*t*(23) = 2.01, *p* = 0.056 *d* = 0.41 [−0.01, 0.82]
Elevation	*t*(20) = 0.70, *p* = 0.490 *d* = 0.15 [−0.28, 0.58]	*t*(17) = 1.10, *p* = 0.286 *d* = 0.26 [−0.21, 0.73]	*****t***(23) = 2.89, ***p*** = 0.008** *****d*** = 0.59 [0.15, 1.02]**
Hope (Herth Hope Index)	*t*(20) = 0.49, *p* = 0.633 *d* = 0.11 [−0.32, 0.53]	*t*(18) = 0.67, *p* = 0.513 *d* = 0.15 [−0.30, 0.60]	*t*(23) = 1.19, *p* = 0.246 *d* = 0.24 [−0.17, 0.65]
Hope—Pathways (Adult Hope Scale)	*t*(21) = 0.36, *p* = 0.724 *d* = 0.08 [−0.34, 0.49]	*t*(18) = 0.87, *p* = 0.397 *d* = 0.20 [−0.26, 0.65]	*t*(23) = 1.35, *p* = 0.191 *d* = 0.28 [−0.14, 0.68]
Hope—Agency (Adult Hope Scale)	*t*(20) = 1.56, *p* = 0.134 *d* = 0.34 [−0.10, 0.78]	*t*(18) = 0.55, *p* = 0.591 *d* = 0.13 [−0.33, 0.58]	*****t***(23) = 3.14, ***p*** = 0.005** *****d*** = 0.64 [0.20, 1.08]**
Nature connectedness (Inclusion of Nature in Self)	*t*(20) = 0.81, *p* = 0.428 *d* = 0.18 [−0.26, 0.61]	*t*(18) = 0.49, *p* = 0.630 *d* = 0.11 [−0.34, 0.56]	*****t***(23) = 2.23, ***p*** = 0.036** *****d*** = 0.46 [0.03, 0.87]**
Nature connectedness (Connectedness to Nature Scale)	*t*(21) = −1.24, *p* = 0.230 *d* = −0.26 [−0.69, 0.17]	*t*(18) = 1.21, *p* = 0.243 *d* = 0.28 [−0.19, 0.73]	*t*(21) = 0.92, *p* = 0.368 *d* = 0.20 [−0.23, 0.62]

Bolded numbers indicate significance.

At the end of the study, participants reported how much time they had spent in nature over the course of the 2 weeks. Participants in the Built condition reported spending the most time in nature (*M* = 50.83, SD = 39.42), followed by those in the Nature group (*M* = 41.67, SD = 40.18), and participants in the Delay group (*M* = 37.50, SD = 33.55). Again, sample size was too small to provide sufficient power for meaningful between-group statistical analyses.

## Qualitative Findings

Combined, participants in the Nature and Built conditions submitted a total of 609 photos (an average of approximately 12 photos per participant). Participants in the Built condition submitted a total of 295 photos of human-built objects and scenes. In addition to these built photos, participants also submitted a total of 46 nature photos. Indeed, 76% of participants in the Built condition submitted at least one nature photo. Participants in the Nature condition submitted a total of 314 nature photos; thus, a total of 360 nature photos and 249 built photos were submitted.

While many participants in the Built condition focused on singular objects in their home, others were mindful of human-built objects outdoors such as houses and buildings, construction sites, outdoor lighting displays, public signs, national flags, and seats on transit. Not surprisingly, most participants in the Nature condition remarked on nature they encountered outdoors, although a few participants were mindful of nature indoors such as house plants or flowers. The nature photos were a mix of singular objects (such as a single bird, leaf, or tree stump) and broader landscapes or vistas.

A description of the emotions that had been evoked by the object/scene accompanied each photo. Some descriptions were quite simple and merely consisted of a list of emotions (e.g., “*amused, focused, productive and engaged,*” “*hope serenity,*” “*disgust,*” “*uncertain, anxious, jumpy*”). However, the bulk of entries (69%) were one-to-three-sentence paragraphs providing detailed, explanatory descriptions. For example:

“*Gratitude, joy, and pride. Grateful to have these plants to tend to and grateful for how these plants are contributing to a healthier living space; joy to see something green and alive as well as them thriving even during a harsh winter; and pride at how I have been keeping these plants alive*” - Participant jpvt4234 [Nature photo]

“*Love, comfort, peacefulness, satisfaction, joy. I purchased this chair over twenty years ago when I bought my first home and was still single. It has been “my spot” for reading, reflection and prayer… and, later, when nursing my little ones. So many memories connected to it!*” - Participant jcad5225 [Built photo]

Responses were first coded for valence as either positive or negative.^2^ Positive and negative themes were evident in descriptions of both nature and built photos. For example:

“*The bunny prints evoke a sense of lightness and makes me smile.*” - Participant hrgp1498 [Nature photo]

“*Slightly sad as the sun is still low on the horizon, and the sky is a tepid blue.*” - Participant hfco6313 [Nature photo]

“*given by my partner. love the stained glass look. it makes me happy and feel appreciated.*” - Participant jtud5283 [Built photo]

“*Lamps are So annoying take so much space wires all over… annoyed with it is what i feel*” - Participant jkqs9562 [Built photo]

**Figure 1:**
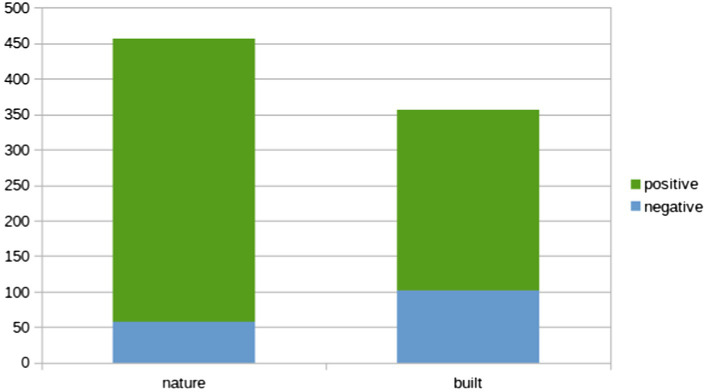
Ratios of positive and negative emotions reported for nature vs. built photos.

Across photo type, the ratio of positive to negative emotions evoked was 5.1:1 (or about 83% positive emotions, 17% negative emotions). However, the ratio of positive to negative emotions differed by photo type. The ratio of positive to negative emotions reported for nature photos (8:1) was over two-and-a-half times the ratio of positive to negative emotions reported for photos of human-built objects (3:1; see Figure 1). Chi-square analysis and examination of the adjusted standardized residuals in the contingency table revealed that photo type (nature vs. human-built) had a significant impact on emotional valence *X*^2^(1, *N* = 813) = 22.91, *p* < 0.001, *V* = 0.17. Negative emotion themes were significantly more likely to be associated with built photos (*z* = 4.8) than with nature photos; indeed negative emotion themes were 2.13 times more likely to be associated with built photos than with nature photos, RR = 2.13, 95% CI [1.55, 2.93].

**Figure 2:**
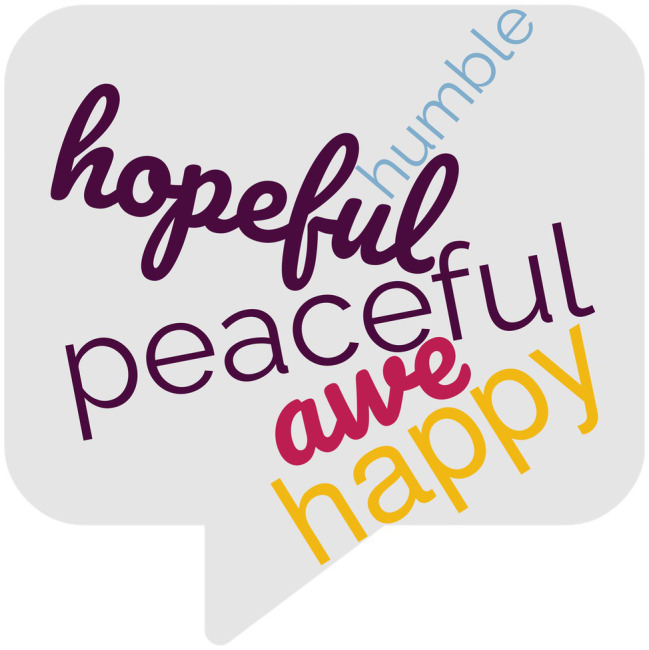
Wordcloud graphic depicting emotion themes most likely to be associated with nature photos.

**Figure 3:**
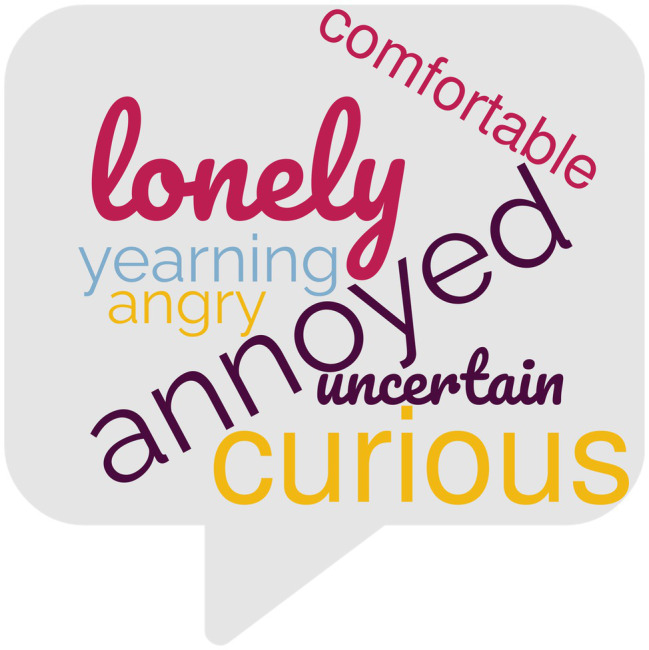
Wordcloud graphic depicting emotion themes most likely to be associated with built photos.

Responses were then coded for emotion themes; 17 positive (e.g., happy, peaceful, awe, grateful, vibrant) and 12 negative (e.g., sad, anxious, annoyed, lonely) themes emerged (see Tables 4 and 5 for complete list of themes) reflecting a varied array of emotions. Chi-square analysis revealed that there was a significant association between photo type and emotion theme *X*^2^(26, *N* = 813) = 114.45, *p* < 0.001, *V* = 0.38. See Figures 2 and 3 for wordcloud graphics illustrating the emotion themes most likely to be associated with nature and built photos.

**Table 4:** Positive emotion themes from content analysis.

Theme	Emotions included in theme	Counts		
		Total	Built	Nature
Happy	Joy, glad, light, amused, positive, good, mirth, thrilled	189	71	118
Peaceful	Peaceful, serene, content, relaxed, tranquil, pleased, nice, quiet, composed, mellow	119	27	92
Awe	Wonder, amazed, fascinated, admiration, respect, vast	58	13	45
Grateful	Appreciative, blessed, privileged, cherish, honoured, lucky	47	23	24
Vibrant	Exhilarated, energetic, growth, alive, excited, playful, recharged, giddy, childlike, festive, lively, youthful	37	16	21
Curious	Intrigued, surprised, adventurous	35	25	10
Connected	Unity, kinship, loved, embraced, community	34	17	17
Comfortable	Warm, cozy, like home, familiar, satisfied	27	17	10
Strong	Powerful, resilient, adaptable, endurance, determined, ambitious, productive, accomplished, perseverance	27	13	14
Hopeful	Optimistic	22	6	16
Safe	Protected, sheltered, relief, friendly, kind, welcome, cared for, caring	17	9	8
Inspired	Creative, imaginative	13	4	9
Thoughtful	Reflective, focused, alert	11	6	5
Free	Released, cathartic, independent, open	8	4	4
Proud	Clever	7	5	2
Humble	Insignificant, small, simple	4	0	4
		655	256	399

**Table 5:** Negative emotion themes from content analysis.

Theme	Emotions included in theme	Counts		
		Total	Built	Nature
Sad	Melancholy, unhappy, down, blue, gloomy, dreadful, disheartened, dark, gross, depressed, solemn, dull	43	20	23
Anxious	Concerned, pensive, trepidation, apprehensive, overwhelmed, on edge, uncomfortable, distressed, uneasy, jumpy, worried	26	13	13
Yearning	Nostalgic, wishful, longing, remembering, wistful, regret	22	16	6
Annoyed	Resentful, trapped, frustrated, impatient, imperfect, inept, disappointed, irritated, restricted, exasperated	21	19	2
Lonely	Separated, forgotten, neglected	13	12	1
Uncertain	Conflicted, irritated, divided, confused, cluttered, unsure, hesitant, puzzled	11	9	2
Cold	Barren, sterile	6	3	3
Anger	Disgust	6	5	1
Fear	Terrified, vulnerable	5	1	4
Weary	Tired	3	1	2
Rushed	Busy	2	2	0
		158	101	57

Nature photos were significantly more likely to be associated with the emotion themes of feeling peaceful (*z* = 5.0), awe (*z* = 3.4), and happy (*z* = 2.0). Examples of such entries are:

“*A feeling of peace and tranquillity. This is a tree in my backyard. I always love hearing birds and squirrels from this tree as it helps calm me.*” - Participant nbnk4742

“*I felt awe that such a tiny bird can withstand the cold.*” - Participant hurv5948

“*They are very difficult to see, but there are about 6 or 7 little "tweety birds" as I call them, sitting in this lilac bush, singing their songs. Seeing and hearing them always makes me happy!*” - Participant bbjz4952

Although not reaching significance level, nature photos were also more likely to be associated with themes of feeling humble (*z* = 1.8),

“*I love trees, especially big ones. I feel humbled by their size, knowing just how much more lies beneath the surface too.*” - Participant bebk8552

“*The big blue sky and the trees that have stood there for years in all weather made me feel small and insignificant, but in a good way.*” - Participant jtyr8639

and hopeful (*z* = 1.6).

“*This barren tree reminded me that this pandemic, though seemingly never-ending, will eventually come to an end, just like this Tamarack tree will eventually grow back its leaves. This made me hopeful.*” -hzme1309

Built photos were significantly more likely to be associated with the emotion themes of feeling annoyed (*z* = 4.4), lonely (*z* = 3.5), curious (*z* = 3.5), yearning (*z* = 2.8), uncertain (*z* = 2.6), anger (*z* = 2.0), and comfortable (*z* = 2.0). Examples of such entries are:

“*Frustration, annoyance, irritation -- why do computers not work easily and intuitively?!*” - Participant bypu4249

“*Forgotten. Alone. Fringe. Isolation. Part of society, but not. Avoidance.*” - Participant jjvh9725

“*curiousity and confusion. i was curious when i saw this sign in a school yard and went to check it out but then i was confused by it cause i dont know what it’s for??*” - Participant neba2957

“*This weekend I sorted through many kitchen and decorative items that were my mom’s (she died two years ago). Coming across her favourite little pot that she used all the time while growing up made me really miss her and wish we could have a visit!.*” - Participant hrkk9270

“*Sad, happy, angry. Mixed emotions. When I see the scale, it reminds me of all the days I’ve been saddened or happily surprised by the numbers shown. I’m angry at myself for being influenced so easily by the numbers.*” - Participant jmwp2548

“*Long day. Cozy bed. Welcome relief, relaxation, and a feeling of warm and safe comfort with no screens in sight.*” - Participant jmrk2958

**Figure 4:**
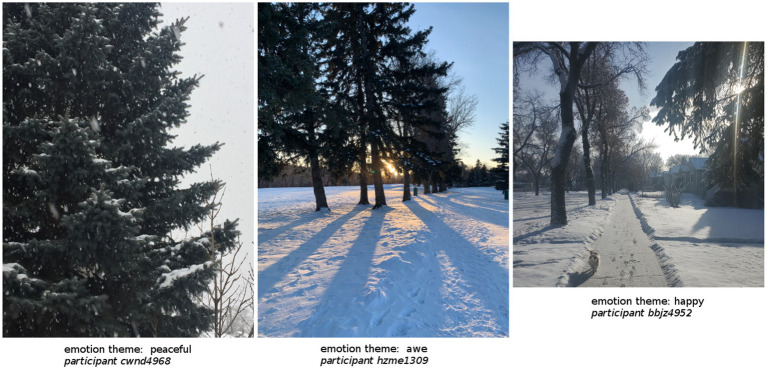
Selection of photos as illustrative sample of the top three emotion themes most likely to be associated with nature photos.

**Figure 5:**
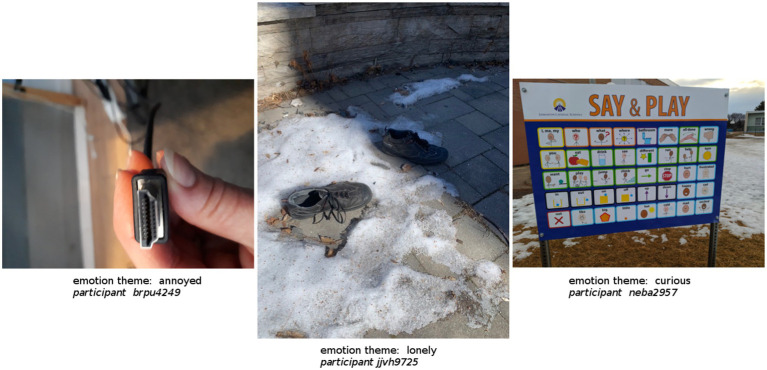
Selection of photos as illustrative sample of the top three emotion themes most likely to be associated with built photos.

See Table 6 for complete chi-square analysis. See Figures 4 and 5 for a selection of three photos from the Nature condition and three photos from the Built condition that serve as illustrative samples of the top three emotion themes most likely to be associated with each type of photo.

**Table 6:** Chi-square analysis for emotion themes by photo-type.

Adjusted standardized residuals indicating significance of positive association with photo-type			
Theme	Built photos	Theme	Nature photos
Annoyed	**4.4**	Peaceful	**5.0**
Lonely	**3.5**	Awe	**3.4**
Curious	**3.4**	Happy	**2.0**
Yearning	**2.8**	Humble	1.8
Uncertain	**2.6**	Hopeful	1.6
Anger	**2.0**	Fear	1.1
Comfortable	**2.0**	Inspired	1.0
Rushed	1.6	Weary	0.4
Proud	1.5	Vibrant	0.1
Safe	0.8		
Thoughtful	0.7		
Connected	0.7		
Grateful	0.7		
Anxious	0.6		
Strong	0.5		
Sad	0.4		
Cold	0.3		
Free	0.3		
	*X*^2^(26, *N* = 813) = 114.45, *p* < 0.001, *V* = 0.38		

Bolded numbers indicate significance.

### Post-intervention Comments

At the end of the study, participants in the Nature and Built conditions were asked if they had learned anything from participating in the study. Almost all these participants responded with a comment about their experiences in the study (86%). Across both conditions, participants reported that engaging in the study made them more aware, and more appreciative, of their surroundings. In the Built condition, several participants commented on how they were reminded of the joy they get from items they chose to keep around.

Nature was mentioned by just over half of the participants (9 of 16) in the Built condition. These comments reflected either a greater affinity for natural compared to human-built objects,

“*I tend to be paying more attention to my surroundings when I am in a more natural setting.*” - Participant brvp7389

“*I found it hard to take pictures of human made things. I’m more drawn to photograph nature and living things - so I found it a bit frustrating.*” - Participant ncfp4768

or reflected how paying attention to human-built objects reinforced their appreciation for nature

“*I have more appreciation for man-made objects that make our lives easier but even more appreciation for Earth since the materials for these objects are extracted from the very earth I live on.*” - Participant jmwp2548

Concern for nature surfaced in comments from participants in both conditions. For example:

“*This study reinforced my negative feelings towards new builds on sites that could be left natural.*” [- Participant vhfq5974, Built condition]

“*I get great comfort from my awareness of the “circle of life” and my place in that, but I feel that humans have done more harm than good.*” [- Participant jtyr8639, Nature condition]

Several participants in the Nature condition noted how the study reaffirmed the importance of the natural world in their lives, in particular everyday nature.

“*Last week was a pretty tough week as I got news that I may be laid off from my job, but being in nature helped calm me and keep things in perspective.*” - Participant vbvp9673

“*This study reinforced the importance of spending time connecting with the natural world to me. It also reinforced the importance of paying attention to everyday delights and that as someone who lives in a city, feeling connected to plants and animals is still possible even in a more metropolitan environment. All I have to do is spend time outside and observe and savour.*” - Participant jpvt4234

Others comments expressed new learning about their relationship with nature,

“*being in nature is fulfilling*” - Participant jfhg8792

“*Humans need to be in nature!*” - Participants cwnd4968

“*I also realized that I rarely just appreciate a natural object or organism. I want to know what it is, how it got there, what it is doing*” - Participant jtyr8639

“*My appreciation of nature involves my human interaction with it, whether it be skiing, cycling, walking, or sitting on our deck appreciating the blue skies and the return of birds to our surroundings.*” - Participant hwgr6368

insights regarding the ease of access to nature, “*I learned that there is lots of nature around*” or their discovery of new aspects of nature:

“*I learned how to be more attentive of the sounds around me, which then helped me to see more living things. I also found an appreciation for the way things grow, like different trees and plants, and serve homes for different animals. It just made me feel like nature is so perfect and it doesn’t even have to try. It just is.*” - Participant jjzr9324

Taken as a whole, these qualitative findings support the quantitative results in which participants in the Nature condition (but not those in the Built or Delay condition) reported significantly higher levels of well-being, hope agency, and connectedness to nature at post- compared to pre-intervention.

## Discussion

In the current 2-week study, we tested the efficacy of the Noticing Nature Intervention to boost various dimensions of wellbeing (including hope) during winter months in a sample of community adults. As per Passmore and Holder’s (2017) original study testing this nature-based wellbeing intervention, participants were randomly assigned to one of three conditions: the Noticing Nature Intervention, a parallel condition involving noticing what emotions were elicited by everyday human-built objects/scenes, or a “Delay”/business-as-usual condition. Although the current study was under-powered, and thus caution is needed when interpreting these results, preliminary analyses indicated that participants who engaged in the Noticing Nature Intervention reported significantly higher levels of several dimensions of wellbeing at post-intervention compared to pre-intervention: positive affect, satisfaction with life, elevation, and hope agency, along with nature connectedness and marginally significantly higher levels of transcendent connectedness. Effect sizes (*d*s from 0.41 to 0.64) were in the upper-range of the average effect size of other positive psychology interventions (*d*s from 0.20 to 0.61) reported in meta-analyses (Sin and Lyubomirsky, 2009; Bolier et al., 2013; Weiss et al., 2016; Chakhssi et al., 2018; Hendriks et al., 2018, 2020; Carr et al., 2020; van Agteren et al., 2021). No significant differences emerged for any variable at post-intervention compared to pre-intervention for those in either of the two control groups.

Participants in the Nature and Built groups provided photos of the natural or human-built objects (dependent on random assignment) which evoked emotions in them; these photos were accompanied by brief descriptions of how these aspects of their everyday environments made them feel. While both positive and negative emotions were evoked by everyday nature and everyday built objects/scenes encountered, the ratio of positive to negative emotions differed by photo type. For nature photos, the ratio of positive to negative emotions was 8:1, while the ratio of positive to negative emotions for photos of human-built objects was 3:1, thus, dovetailing with the boosts to wellbeing indicated by the quantitative results. Positive emotion themes of peace/calm, awe, and happiness were significantly more likely to associated with nature photos than with built photos. Negative emotion themes of annoyance, loneliness, yearning, uncertainty, and anger more significantly more likely to be associated with built photos than with nature photos.

### Comparison of Findings to the Previous Noticing Nature Intervention Studies

As noted above, Passmore and colleagues’ (Passmore and Holder, 2017; Passmore et al., 2022) previous studies testing the efficacy of the Noticing Nature Intervention as a well-being intervention were conducted during relatively clement weather in early fall/autumn months, while the current study was conducted in colder, winter months. Nonetheless, both quantitative results and qualitative findings in the current study paralleled those in Passmore and colleagues’ previous two studies. In all three studies (current and previous two), engaging in the Noticing Nature Intervention significantly boosted various aspects of well-being. In all three studies, participants randomly assigned to the Noticing Nature Intervention did not report spending more time in nature over the course of the study than did participants in other conditions.

In all three studies, negative emotions were significantly more likely to be associated with built (vs. nature) photos (2017: *z* = 6.2, 2021: *z* = 7.0; current: *z* = 4.8). The positive emotion themes of “peaceful/calm” and “awe” emerged in all three studies as being significantly more likely to be associated with nature (vs. built) photos (2017: *z*_peace_ = 4.8, *z*_awe_ = 3.6; 2021: *z*_calm_ = 7.1, *z*_awe_ = 2.0; current: *z*_peace_ = 5.0, *z*_awe_ = 3.4); while the negative emotion theme of “annoyance” (or the related theme of “anger”^3^) emerged in all three studies as being significantly more likely to be associated with built (vs. nature) photos (2017: *z*_annoyed_ = 5.2; 2021: *z*_angry_ = 5.2; current: *z*_annoyed_ = 4.4).

Post-study comments in both the original (Passmore and Holder, 2017) and current study were similar.^4^ In both studies, participants in the Nature condition noted how engaging in the Noticing Nature Intervention reaffirmed for them how important nature was to their wellbeing. In both studies, comments from a number of participants in the Built condition (one-third in the 2017 study, just over half in the current study) included references to nature. In particular, these comments referred to how people were more emotionally drawn to natural than human-built objects.

In the current study, comments from participants in the Built condition also included references to enhanced feelings of joy from their cherished (built) objects in their homes. Such comments were not evident in the original (Passmore and Holder, 2017) study. This may be a function of the different sample populations across these two studies. In the original study, participants were younger undergraduate university students with a mean age of 20.09 years. Many university students reside in somewhat sparse or less-personal living spaces away from what they would consider “home.” Participants in the current study, however, were community adults with a mean age of 46.75. Not only were these participants residing in their own homes, simply by virtue of their age they would have had vastly greater opportunity than students to accumulate cherished items associated with life memories and experiences. It is also possible that participants in the current study spent more time in their homes than did participants in the previous study, and thus, would have had more time to appreciate the built objects in their homes. If this were the case, greater time spent at home may have been due to life circumstances (e.g., retired, jobs that allow or working at home) or due to inclement winter weather. Future research is needed to disentangle these factors.

### Limitations and Future Directions

As with all studies, the current study had limitations. Due to unexpected difficulties recruiting community participants, our sample size was quite small, yielding insufficient power for truly meaningful quantitative analyses, as noted above. Nearly one-third of participants dropped out before completing the study. While this is not entirely unusual in experimental studies of this length of time, it is possible that this may have skewed the data in favor of those more nature affiliated. Of the 26 participants who did not complete the 2-week study, 12 had been randomly assigned to the Human-Built condition, 5 to the Delay condition, 6 to the Nature condition, and 3 dropped out before being assigned to a condition. Sample size is too small to meaningfully conduct analyses examining if initial levels of nature connectedness differed significantly between those who dropped out of and those who completed the study.

Our sample consisted of an uneven gender ratio of respondents (78% female), all of whom were from North America. Results from recent work of Passmore et al. (2022) evidenced that the Noticing Nature Intervention was effective at boosting wellbeing in a sample of primarily male (64%) undergraduates at a university in China. Nonetheless, more work is needed in this area to determine any potential differential effects across sexes, cultures, and age groups.

Our sample in the current study was recruited from more than one city and geographic area. Although the majority (66%) of participants were from Edmonton, Alberta, the remaining 34% were from cities in other parts of Canada and the northern United States. Although it was winter in all locations, colder, snowier weather was more prevalent in some cities than others during the period of the study. Additionally, signs of spring may have been more evident (depending on location) to participants who engaged in the study in the first 2 weeks of March compared to those who engage in the study during January or February.

We recommend that future research assessing the efficacy of engaging in a nature-based wellbeing intervention during winter months utilize a larger sample of participants from only one city, in order to more closely control for these possible confounds. We also recommend that future studies collect data only during the heart of winter, when people are most likely to need the wellbeing boost that nature provides. Limitations notwithstanding, quantitative and qualitative results in the current study were in line with results from previous Noticing Nature Intervention studies (Passmore and Holder, 2017; Passmore et al., 2022).

## Conclusion and Implications

Results of the current 2-week study suggest that engaging in the Noticing Nature Intervention (i.e., noticing the everyday nature around you and the emotions it evokes) can provide a wellbeing boost, even in the cold of winter. These results make a unique contribution to the current literature in that this study is the first (that we are aware of) to test any nature-based wellbeing intervention during colder, winter months, and to directly assess the impact of a nature-based wellbeing intervention on levels of hope. Results of the current study also bolster previous findings (Passmore and Holder, 2017; Passmore et al., 2022) regarding the efficacy of the Noticing Nature Intervention to enhance wellbeing, and are in line with previous findings that these wellbeing boosts do not appear to be a result of spending more time in nature, but rather of simply noticing the everyday nature encountered in one’s daily routine.

Too often, we ignore the nature around us—the tree at the bus stop, the bird in our backyard. This is particularly so in the winter, which brings its own beauty, peace, joy, and even hope. We *can* access everyday nature in the winter as a pathway to enhancing wellbeing; we just need to notice it. As this participant wrote “*Just noticing the sparkles in the snow made me feel appreciative that we get to experience winter*” (participant hurv5948).

## Data Availability Statement

The datasets presented in this study can be found in online repositories. The names of the repository/repositories and accession number(s) can be found at: https://osf.io/s6y5e/?view_only=33d673d4f7c04fa89577f70be82b037c.

## Ethics Statement

This research was approved by the Research Ethics Board of Concordia University of Edmonton (004-2020-12-16-HP). The patients/participants provided their written informed consent to participate in this study.

## Author Contributions

H-AP performed the design and running of the research study, all data analysis, and writing of article. AY contributed to the coding of qualitative data, preliminary draft of qualitative findings, and write–up. JB performed the coding of qualitative data. All authors contributed to the article and approved the submitted version.

## Funding

This work was supported by a Seed Grant from Concordia University of Edmonton (CRG-SEED-2010-01). This grant ($4,997.00 CAD) funded subscription to SurveyMonkey. An undergraduate Research Assistant, and the fifty $50 Amazon e-vouchers used as a draw to compensate participants.

## Conflict of Interest

The authors declare that the research was conducted in the absence of any commercial or financial relationships that could be construed as a potential conflict of interest.

## Publisher’s Note

All claims expressed in this article are solely those of the authors and do not necessarily represent those of their affiliated organizations, or those of the publisher, the editors and the reviewers. Any product that may be evaluated in this article, or claim that may be made by its manufacturer, is not guaranteed or endorsed by the publisher.

## Footnotes

^1^g*Power sample size estimates: ANCOVA: *f* = 0.25; α error = 0.05; power = 0.80; numerator df = 2; groups = 3; covariates = 1, *N* = 158; paired t-test: *dz* = 0.50; α error = 0.05; power = 0.80; *N* = 34; between-group t-test: *d* = 0.50; α error = 0.05; power = 0.80; allocation ratio = 1; *N* = 128.

^2^Many descriptions reflected more than one theme; therefore, each description was categorized and counted in more than theme.

^3^The Passmore et al. (2022) study utilized a sample from China. Nuanced grouping of specific emotion words were thus, somewhat different in that study compared to the 2017 and current study.

^4^Post-intervention comments were not solicited in the Passmore et al. (2022) study.
